# 

**Published:** 1895-05

**Authors:** 


Albert Lynch, whose work is becoming so much more generally known to Americans through his drawings in *Scribner's Magazine* and his cover designs for *The Ladies' Home Journal*, is a Peruvian by birth, but of English parentage. He is thirty-three years of age, unmarried and lives in Paris. He commands the highest prices for his work, his smallest water-color paintings readily selling for $600 to $900 each. In 1893 he received the salon's first prize for his beautiful panel of "Spring," showing a single figure. Recently *The Ladies' Home Journal* acquired all publication rights to this painting, and it will serve as one of the cover designs for that magazine. The May issue of the *Journal* also has a design by Lynch, portraying his conception of a woman's ideal costume. A succession of other cover designs by Lynch will follow these two.

---

Medicine is the name of a monthly journal of medicine and surgery edited by Dr. Harold N. Moyer, of Chicago, that made its appearance in April, 1895. The first number is a handsome specimen of journalistic work and reflects much credit upon its accomplished editor. It is published in Detroit by George S. Davis, the well-known medical publisher. With Dr. Moyer and Mr. Davis associated interestedly in the magazine its success is already assured.

The April number of *Germania* completes its sixth volume and is accompanied with a complete index of its contents. We cannot too highly commend this magazine and would earnestly urge every student and teacher of the German language to subscribe for it. The perusal of its index will convince everyone that it is a treasure beyond price.

Volume VI. of this magazine contains eight standard German novels, carefully annotated and prepared for American students by eminent professors of German. It also contains about fifty fables and short stories by standard authors, supplemented by interlinear translations or complete vocabularies and notes. Numerous articles on grammar, literature, synonyms, pronunciation and spelling by well-known teachers of German are found in every number. It is the opinion of some of our leading college professors that *Germania* is the best effort yet made to assist the student of German and to interest him in his pursuit. The magazine is used as a regular text-book by the professors in the Universities of Chicago, California, Michigan, South Carolina, Syracuse, Northwestern University, etc., and in numerous normal and high schools. It is published by the Germania Publishing Co., of Boston, Mass. Subscription price, $2.00.

---

The medical journal heretofore known as *Food* has changed its name to *The Journal of Practical Medicine.* It has also put itself into a new dress and presents, in its April issue, an improved appearance in every way.

---

The Rio Chemical Company, of St. Louis, has issued and sent out to physicians an excellent map of the United States, suitable to have on an office wall. It also gives statistical information concerning the civil divisions of the world arranged by continents. Any physician who has not received one of these useful maps should address the publishers as above.

---

Annales de l'Insitut Ste. Anne is the name of a new medical publication conducted by Dr. C. Jacobs, president of the Belgium Gynecological and Obstetrical Society and an honorary fellow of the American Association of Obstetricians and Gynecologists. It is published quarterly at Brussels and Number 1, Volume I., bears date of March 15, 1895.

Dr. Aug. C. Bernays, of St. Louis, has published an illustrated brochure, entitled Report of a Surgical Clinic. It contains the report of six or seven operations made by the author in the presence of members of the Mississippi Valley Medical Association en route to and from the Hot Springs meeting in November, 1894. Some of the cases involved unusual surgical procedures that tax a surgeon's skill and resource. Dr. Bernays is to be congratulated on his results in the series herein published.

